# Effects of the performance management information system in improving performance: an empirical study in Shanghai Ninth People’s Hospital

**DOI:** 10.1186/s40064-016-3436-2

**Published:** 2016-10-13

**Authors:** Yinghui Cui, Zhengyi Wu, Yao Lu, Wenzhong Jin, Xing Dai, Jinxi Bai

**Affiliations:** Department of Shanghai Ninth People’s Hospital, Shanghai JiaoTong University School of Medicine, No. 639, Zhizaoju Road, HuangPu District, Shanghai, 200011 China

**Keywords:** Hospital, Clinical departments, Performance management, Information system, Empirical study

## Abstract

Improving the performance of clinical departments is not only the significant content of the healthcare system reform in China, but also the essential approach to better satisfying the Chinese growing demand for medical services. Performance management is vital and meaningful to public hospitals in China. Several studies are conducted in hospital internal performance management, but almost none of them consider the effects of informational tools. Therefore, we carried out an empirical study on effects of using performance management information system in Shanghai Ninth People’s Hospital. The main feature of the system is that it provides a real-time query platform for users to analyze and dynamically monitor the key performance indexes, timely detect problems and make adjustments. We collected pivotal medical data on 35 clinical departments of this hospital from January 2013 until December 2014, 1 year before and after applying the performance management information system. Comparative analysis was conducted by statistical methods. The results show that the system is beneficial to improve performance scores of clinical departments and lower the proportion of drug expenses, meanwhile, shorten the average hospitalized days and increase the bed turnover rate. That is to say, with the increasing medical services, the quality and efficiency is greatly improved. In a word, application of the performance management information system has a positive effect on improving performance of clinical departments.

## Background

Over the past decades, the healthcare reform in China has already achieved some profound success. The new comprehensive and deepening reform plan, launched in 2013, marked the explicit goals to accelerate the public hospital reform, fulfill government responsibility and establish scientific and effective medical performance management system (Wong et al. 2016; Yip et al. 2012; Blumenthal and Hsiao 2005; Xin 2015; Cheng 2015). Therefore, performance management is vital and meaningful to public hospitals in China, and the critical challenge for them is how to improve the performance of clinical departments that is essential to ensure they are providing high-quality and efficiency medical services.

Several studies are done in hospital internal performance management, but nearly no studies take into consideration the effects of informational tools. With the rapid development of healthcare information technologies and the comprehensive construction of hospital information systems, plenty of hospitals in China built the medical big data information integration platform with clinical data repository (CDR) as the core component, thus achieving data integration, information sharing and cooperative processing of multiple information systems in hospital (Mezghani et al. 2015; Lin et al. 2012; Acampora et al. 2013; Blumenthal 2009; Jiang et al. 2014). Meanwhile, establishing performance management information system (PMIS) based on this medical big data information integrated platform to help supervise and appraisal performance of clinical departments.

So, does the application of PMIS facilitate improved performance of clinical departments? This paper takes Shanghai Ninth People’s Hospital as the research object, and describes the construction and functions of PMIS. In particular, we analyzed the medical data and performance scores on 35 clinical departments that before and after applying the intelligent performance management information system, then conclusions are drawn accordingly. Although similar studies might be done in other hospitals in Shanghai, to our best knowledge, Shanghai Ninth People’s Hospital is the first to complete the big data analysis and forecast in advance while the analysis shows abnormity.

The rest of papers are organized as follows: “Performance management information system” section introduces the construction of this system and describes its characteristics and functions in details. The results of medical data analyses are given in “Case study and results” section. Finally, last section gives summary and conclusion of this paper.

## Performance management information system (PMIS)

**Fig. 1:**
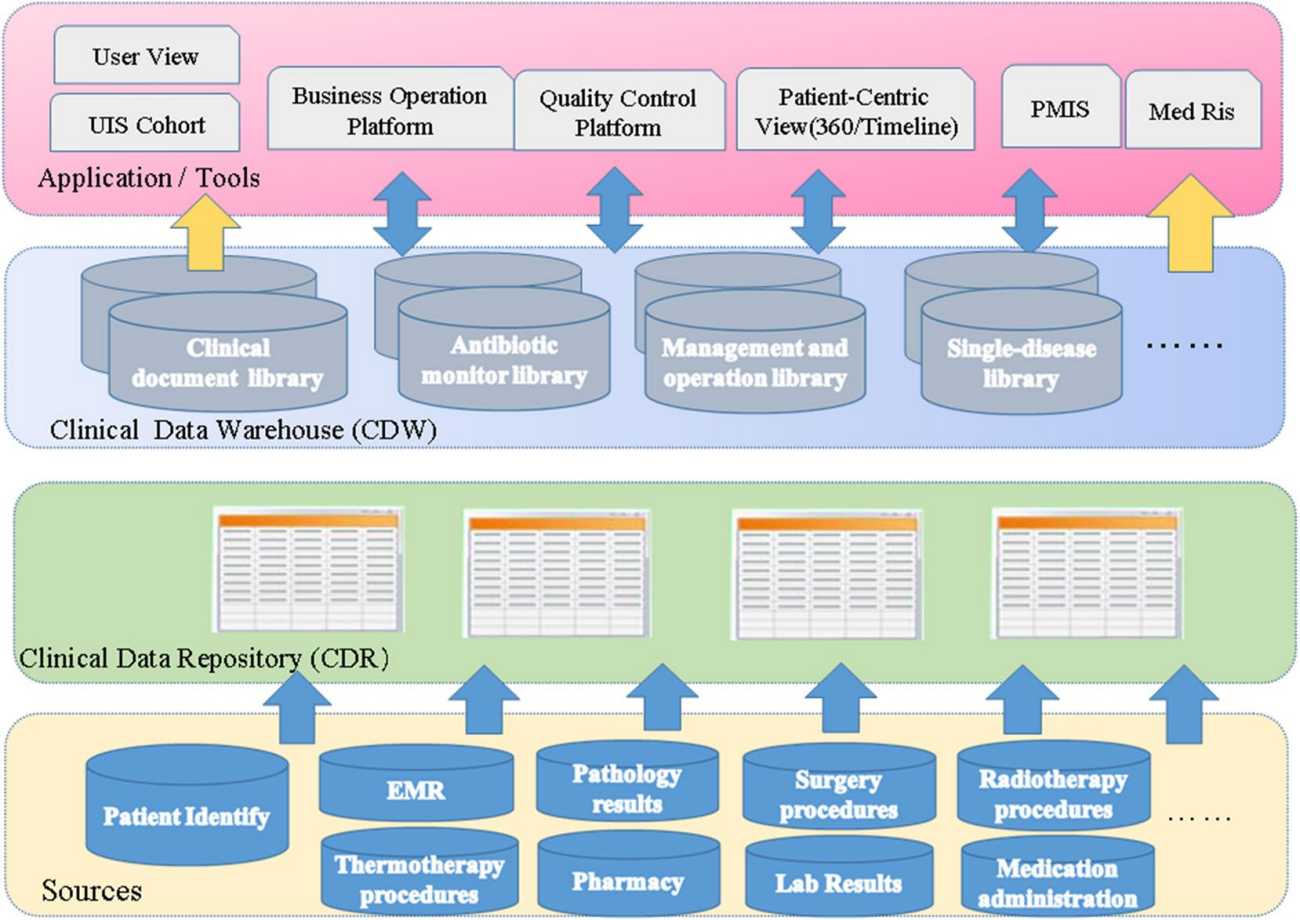
Overall system architecture of the information integration platform

Shanghai Ninth People’s Hospital is a large general hospital integrated with medical treatment, teaching and scientific research, which is characterized by oral medicine and plastic surgery. The hospital contains more than 1000 inpatient beds and 300 dental chairs, thereby allowing for 58,000 hospitalizations, nearly 2.97 million outpatients and emergency patients each year. In July 2013, the hospital cooperated with Microsoft (China) to implement the construction of medical big data information integration platform which takes clinical data repository (CDR) as the core component, and the overall system architecture as shown in Fig. 1.

Because of the history of the development of healthcare information technologies, clinical data are typically stored in relational databases (Yao et al. 2015a). Therefore, the hierarchical structure was designed to integrate various hospital information systems (such as RIS, HIS and PACS, etc.), thus realizing clinical data exchanging, resource sharing, interoperability and unified management of different departments and heterogeneous information systems. The clinical data repository (CDR) is a patient-centric, integrated, real-time, life-long data repository that can aggregate clinical data from multiple sources, and it has stored historical and real-time data from January 2008 until the present and the new data automatically import to CDR every 3 s. On that basis, we built some specific clinical data warehouses (CDW) for our specific purposes to serve on the medical service, clinical study and management of the hospital. Finally, we developed some external applications and tools, such as Business Operation Platform, Quality Control Platform, Patient-Centric View, Medical Research Information System (MedRis) and performance management information system (PMIS).

**Fig. 2:**
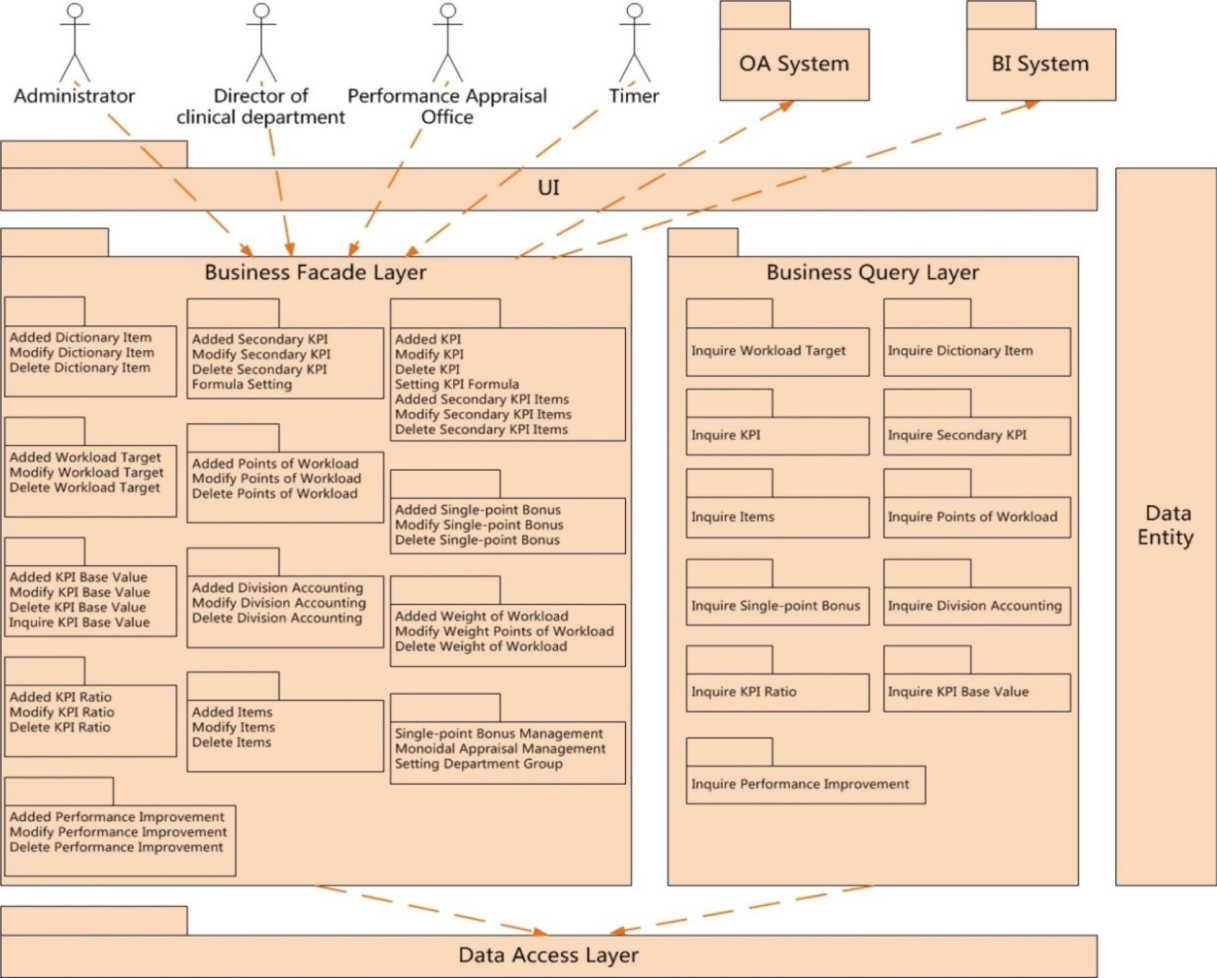
Logical architecture of the performance management information system

Figure 2 describes the logical architecture of the performance management information system (PMIS) which has been used formally in January 2014, and it has six modules: Target Setting and Planning, Performance program, Performance monitoring and improvement, Assessment and Evaluation, Performance Review, System Configuration. User could choose “graph” or “table” to check statistics. The performance management information system (PMIS) could achieve functions as follows:

- Dock with the real business systems, get clinical data from the CDR and automatically calculate the amount of work, the KPI score and bonus distribution of each clinical department. Subjective evaluation data such as patient satisfaction are scored at mobile termination and read automatically to the PMIS through data interface.
- Calculate workload target, KPI base value and ratio of each clinical department automatically and realize the function of accounting and distribution according to the configuration of foundation information.
- Analyze and process basic data, show and export the results in various forms.
- Based on user’s right and limit to classify management. Hospital leader, clinical director and head of Performance Management Office have different system operational permissions.

Results were satisfactory when users were asked about their perceived benefit of applying this performance management information system. The system provides a real-time query platform for users to dynamically monitor the key performance indexes and analyze medical data, thereby, timely find problems and make adjustments. Users are aware of the need to apply the system and they consider it is contributed to provide high-quality and efficiency medical service, and it will clearly facilitate improved performance.

## Case study and results

This section presents the results obtained in the data analysis. To illustrate the effect of performance management information system (PMIS), we collected data on 35 clinical departments in Shanghai Ninth People’s Hospital from January 2013 until December 2014, and divided the data into two groups. The first group data is from January to December in 2013, and the other set of data is from January to December in 2014 after applying the performance management information system. Do comparative analysis between the two sets of data by general statistical description and repetitive measurement deviation analysis with SPSS 20.0 statistical software. All the data in this case study come from the medical big data information integration platform.

### Analysis of the performance scores

Managers of the Performance Management Office calculate monthly performance score of each clinical department according to key performance appraisal indexes which take patient satisfaction, quantity and quality of medical service, grade of disease, control of healthcare expenses and medical professionalism as core contents.

Analyze performance scores by means of repetitive measurement deviation analysis. This case has two repeated measurement factors, time and year. So the result of Mauchly’s test of sphericity gives the Epsilon correction coefficient, and the Greenhouse–Geisser corrected results should be taken (Johnson and Wichern 2007; Huang 2002), as shown in Table 1. Different time levels have significant differences (*P* < 0.05), and the score differences between 2013 and 2014 also have statistical significance (*P* < 0.05).

**Table 1:** Test of within-subjects effects

Measure: MEASURE_1					
Source	Type III sum of squares	*df*	Mean square	F	Sig.
Time					
Sphericity assumed	111.315	11	10.120	5.672	0.000
Greenhouse–Geisser	111.315	7.113	15.649	5.672	0.000
Huynh–Feldt	111.315	9.185	12.119	5.672	0.000
Lower-bound	111.315	1.000	111.315	5.672	0.023
Error (time)					
Sphericity assumed	667.281	374	1.784		
Greenhouse–Geisser	667.281	241.850	2.759		
Huynh–Feldt	667.281	312.301	2.137		
Lower-bound	667.281	34.000	19.626		
Year					
Sphericity assumed	139.626	1	139.626	40.953	0.000
Greenhouse–Geisser	139.626	1.000	139.626	40.953	0.000
Huynh–Feldt	139.626	1.000	139.626	40.953	0.000
Lower-bound	139.626	1.000	139.626	40.953	0.000
Error (years)					
Sphericity assumed	115.919	34	3.409		
Greenhouse–Geisser	115.919	34.000	3.409		
Huynh–Feldt	115.919	34.000	3.409		
Lower-bound	115.919	34.000	3.409		
Time × year					
Sphericity assumed	115.701	11	10.518	5.307	0.000
Greenhouse–Geisser	115.701	6.179	18.726	5.307	0.000
Huynh–Feldt	115.701	7.701	15.025	5.307	0.000
Lower-bound	115.701	1.000	115.701	5.307	0.027
Error (time × year)					
Sphericity assumed	741.290	374	1.982		
Greenhouse–Geisser	741.290	210.069	3.529		
Huynh–Feldt	741.290	261.827	2.831		
Lower-bound	741.290	34.000	21.803		

**Fig. 3:**
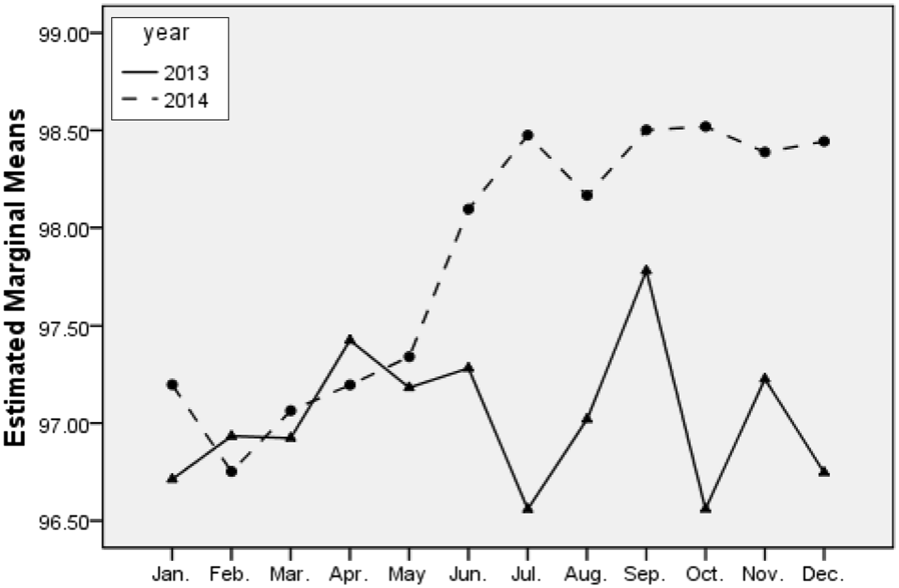
Profile diagram of interaction effect

Combining with Fig. 3, we clearly find the performance scores in 2014 are generally higher than 2013, and the increase of performance scores is statistically significant. It demonstrates that the performance scores are effectively improved when the performance management information system is applied, and the informational tool has a positive effect on improving performance of clinical departments.

### Analysis of the medical service quality and efficiency

To further clarify the influence of this informational tool, a study about the quality and efficiency of medical service was carried out. We analyzed the pivotal medical data on 35 clinical departments from January 2013 until December 2014, 1 year before and after applying the performance management information system.

Descriptive statistics of the medical service are given in Table 2. Compared with 2013, medical service quantity of 2014 maintained continuous rapid growth. The number of outpatients and emergency patients has rapidly increased to over 2.67 million, and the average annual growth rate is 9.68 %. Meanwhile, the number of inpatients was 4.96 % higher than in 2013. Especially the number of surgery has a more than 30 % jump over the same period in 2013.

**Table 2:** Descriptive statistics of the medical service

Variable	2013			2014			Annual growth rate (%)
	Mean	SD	Total	Mean	SD	Total	
Number of outpatients and emergency patients	69,747.89	47,940.729	2,441,176	76,498.8	54,980.618	2,677,458	9.68
Number of inpatients	2359.36	1585.723	51,906	2476.27	1656.776	54,478	4.96
Number of surgery	4232.87	7836.144	135,452	5505.72	10,312.137	176,183	30.04

**Fig. 4:**
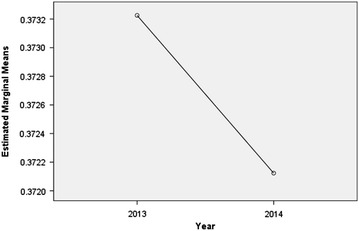
Profile diagram of drug cost proportion

With the deepening reform of healthcare system in China, controlling the proportion of drug cost accounting for total medical expenditure has become a significant objective and great challenge for public hospitals (Liang et al. 2014; Yao et al. 2015b; Song et al. 2014; Yang et al. 2013). Curtailing the drug cost proportion is beneficial to guide the rational medication and control the unreasonable increase of drug expenditure (World Health Organization 2002; Quick et al. 1991; Guan et al. 2011; Le Grand et al. 1999). Figure 4 shows the comparative analysis result of drug cost proportion between 2013 and 2014, and the drug cost proportion decreased after the implementation of the performance management information system in 2014 with statistical significance (*P* *<* 0.05). It revealed that the application of such technology in the hospital performance appraisal had an important significance to keep the high efficiency, which promoted sustainable development of public hospital and better satisfy the people’s growing demand of medical services.

**Fig. 5:**
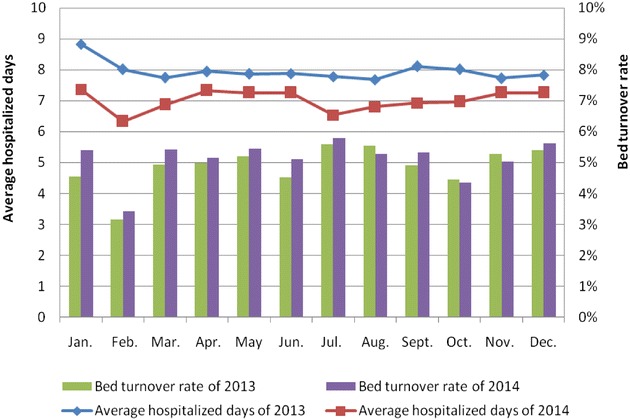
Average hospitalized days and bed turnover rate of surgical departments in 2013–2014

**Fig. 6:**
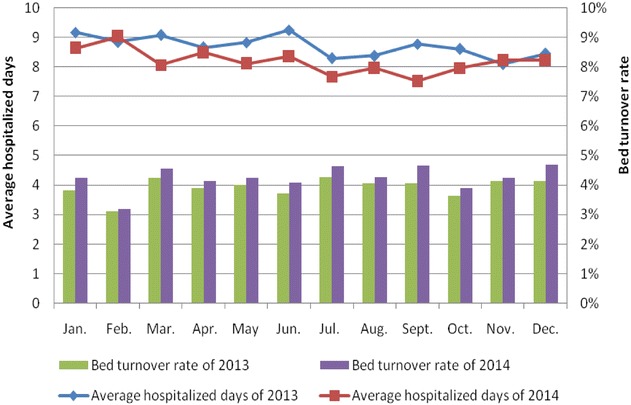
Average hospitalized days and bed turnover rate of non-surgical departments in 2013–2014

Average hospitalized days and bed turnover rate are comprehensive indexes to reflect the medical service efficiency. Shortening the average hospitalized days and increasing the bed turnover rate could effectively improve the performance of clinical departments (Lee and Wang 2006; Tseng et al. 2015; Escuriet et al. 2015; Mullen 2004; Bonfill et al. 2013). For more homogeneity, we analyzed these two indexes of surgical departments and non-surgical departments individually, as shown in Figs. 5 and 6. The x-axis represents month, the main y-axis represents average hospitalized days and the secondary y-axis represents bed turnover rate. As we have seen in Fig. 5, the red line shows the average hospitalized days from January to December of 2014 is completely below the blue line which reflects the average hospitalized days in same period of 2013, and the purple bar represents bed turnover rate of 2014 is nearly all higher than the green bar depicts bed turnover rate of 2013. Almost the same situation is observed in Fig. 6. This led us to draw the conclusion that the average hospitalized days decreased both in surgical departments and non-surgical departments after using the performance management information system, meanwhile, the bed turnover rate had a greater improvement. The result indicates the application of this performance management information system has a practical significance in improving the efficiency of medical service.

## Conclusions

This study represented the constructions and functions of the performance management information system in Shanghai Ninth People’s Hospital that can provide a real-time query platform for users to monitor performance indexes and analyze medical data, thereby, timely detect problems and make adjustments. Additionally, we compared performance scores and crucial medical data on 35 clinical departments of this hospital before and after applying this system. The results show that the performance scores are greatly improved when the performance management information system is used, and the drug cost proportion which could guide the rational medication and control the unreasonable increase of drug expenditure is curtailed. Furthermore, it effectively shortened the average hospitalized days and increased the bed turnover rate. In other words, with the growing demand of medical services, the quality and efficiency of medical service has a significant improvement. Therefore, we could draw a conclusion accordingly that the implementation of performance management information system has a positive effect on improving performance of clinical departments. Long-term assessment for such system is in progress to make the databased more reliable and we anticipate that PMIS system could be implement in other hospitals in the future.
